# Using the Conversation Starter Kit in Canada to Promote Resident Care Planning Discussions in Long-Term Care

**DOI:** 10.1093/geroni/igaa057.796

**Published:** 2020-12-16

**Authors:** Sharon Kaasalainen, Tamara Sussman

**Affiliations:** 1 McMaster University, Hamilton, Ontario, Canada; 2 McGill University, Montreal, Ontario, Canada

## Abstract

Advance care planning (ACP) is still rare in Canadian long-term care (LTC) homes. Residents and their families view ACP as uncomfortable and difficult to implement, leading them to avoid these discussions. The purpose of this study was to explore the perceptions of LTC residents and their families about using an ACP tool called The Conversation Starter Kit. This study utilized a mixed methods approach. Data was collected in four LTC homes in Ontario, Canada from 78 residents and family members. Data was analyzed using thematic analysis and descriptive statistics. All participants read all sections but only 73% completed all sections of the toolkit. Participants spent an average of 52.3 minutes completing the toolkit and 36.4 minutes discussing it with their family members and/or LTC staff. Participants reported: a better understanding of ACP after using the tool (80%), that the tool helped clarify the available resources and/or choices (53%), and that they felt less apprehensive about ACP after using the tool (60%). Qualitative findings revealed many strengths (e.g., usefulness, ability to start difficult conversations, content and clarification), and weaknesses of the tool (e.g., redundant information, difficulty understanding the content and lack of information regarding medically assisted dying). Family members noted that the toolkit would have been helpful to receive earlier on in their family members’ disease trajectory, perhaps before being admitted into LTC. These study findings support the feasibility and acceptability of the tool to engage residents and family members in/; ACP discussions in LTC.

